# Adjuvant capecitabine in women with triple-negative breast cancer with residual disease after carboplatin-containing neoadjuvant chemotherapy

**DOI:** 10.1007/s10549-026-07949-x

**Published:** 2026-03-30

**Authors:** Grazielle Morais Tavares, Susana Oliveira Botelho Ramalho, Leonardo Roberto da Silva, Guilherme Defante Telles, Geisilene Russano de Paiva Silva, Renato Zocchio Torresan, Sophie Françoise Mauricette Derchain

**Affiliations:** 1 Department of Obstetrics and Gynecology, Faculty of Medical Sciences, University of Campinas, Unicamp, Campinas, São Paulo, Brazil; 2 Division of Oncology, Hospital da Mulher Prof. Dr. José Aristodemo Pinotti (CAISM/UNICAMP), University of Campinas (UNICAMP), Campinas, São Paulo, Brazil; 3 Department of Biochemistry and Tissue Biology, Institute of Biology, University of Campinas (UNICAMP), Campinas, São Paulo, Brazil; 4 Faculdade de Ciências Médicas-UNICAMP, Postal code I Street Alexander Fleming, 101. Cidade Universitária, Campinas, São Paulo, 13083-881 Brazil

**Keywords:** Triple-negative breast cancer, Neoadjuvant chemotherapy, Carboplatin, Capecitabine, Pathologic complete response, Real-world study

## Abstract

**Background:**

Triple-negative breast cancer (TNBC) is an aggressive subtype characterized by early relapse and limited therapeutic options. Carboplatin added to anthracycline/taxane neoadjuvant chemotherapy (NACT) raises pathologic complete response (pCR) rates. The role of adjuvant capecitabine following NACT containing carboplatin remains unclear, particularly in real-world settings.

**Methods:**

Data of patients who were diagnosed with TNBC and received NACT at CAISM/UNICAMP (2017–2023), followed up until June 2025 were analyzed retrospectively. The cohort study included 184 women with TNBC treated with anthracycline, taxane, and carboplatin-based NACT. Clinical, pathological, and treatment data were collected from institutional databases and the CAISM Biobank. Survival outcomes were analyzed according to pCR and use of adjuvant capecitabine among patients with non-pCR. Median follow-up was estimated using the reverse Kaplan–Meier method. Additional exploratory analyses included stratification by Residual Cancer Burden (RCB-I vs RCB-II/III), multivariable Cox models including capecitabine as the exposure variable, and administrative censoring sensitivity analyses at 36 and 48 months. Disease-free survival (DFS) and overall survival (OS) were estimated by Kaplan–Meier method and compared with log-rank tests; multivariable Cox models assessed prognostic factors.

**Results:**

Among 184 women, pCR was achieved in 40% and was associated with younger age, earlier clinical stage and high histologic grade. Survival outcomes were significantly better for patients with pCR, with an approximately 85–87% reduction in the risk of recurrence and death compared with non-pCR. Among the 111 patients with residual disease, 44 received adjuvant capecitabine. Adjuvant capecitabine did not improve DFS (HR = 0.96; 95% CI 0.52–1.78) or OS (HR = 0.70; 95% CI 0.33–1.46) in univariable analyses. In multivariable models stratified by capecitabine use, Ki-67 remained the only independent prognostic factor slightly associated with worse DFS, while capecitabine showed no significant effect.

**Conclusions:**

In this real-world cohort, achieving pCR after carboplatin-containing NACT was the strongest determinant of favorable prognosis. Adjuvant capecitabine was not associated with statistically significant improvement in survival outcomes. The role of capecitabine following prior platinum exposure remains uncertain and warrants further investigation.

## Introduction

Triple-negative breast cancer (TNBC) accounts for approximately 13% of breast cancer cases and is characterized by aggressive behavior, high proliferative rate, and high-risk of early relapse. Anthracycline- and taxane-based neoadjuvant chemotherapy (NACT) historically constituted the standard treatment for locally advanced TNBC [1]. More recently, the addition of immune checkpoint inhibitors to neoadjuvant chemotherapy has significantly improved pCR rates and translated into superior event-free and overall survival in early-stage TNBC [2–4]. However, access to immunotherapy remains heterogeneous across health systems, particularly in middle-income countries.

In parallel, strategies aimed at increasing pCR rates, which strongly correlate with improved disease-free survival (DFS) and overall survival (OS), have included the incorporation of carboplatin into standard NACT regimens. Clinical trials such as CALGB 40603, GeparSixto, and BrighTNess showed that adding carboplatin to NACT significantly increases pCR rates in TNBC [5–7] Subsequent meta-analyses have confirmed not only the impact of carboplatin on pCR but also its association with improved DFS and OS [8].

Post-NACT pCR is the strongest predictor of favorable outcomes in TNBC [9]. For patients not achieving pCR, residual disease is a marker of worse prognosis and justifies adjuvant therapy. The CREATE-X study demonstrated that adding adjuvant capecitabine in patients with breast cancer after anthracycline- and taxane-based NACT improved DFS and OS, especially in TNBC [10]. Similar results were observed in SYSUCC-001, which evaluated maintenance of capecitabine and also found significant benefit [11]. Conversely, the GEICAM/CIBOMA study showed no advantage, possibly due to methodological differences such as inclusion of lower-risk patients or use of the drug after adjuvant chemotherapy [12]. Ye et al. (2022) in their meta-analysis reinforced the benefit of capecitabine in TNBC regardless of menopausal or nodal status, although their results are essentially based on the CREATE-X study [13]. Many centers have incorporated adjuvant capecitabine in women with TNBC after NACT, with or without carboplatin, when residual disease is present. However, no clinical trials specifically evaluated capecitabine after carboplatin-containing regimens; available evidence derives from real-world observational studies suggesting heterogeneous and non-consistent benefit in this setting [14–17].

The objective of this study was to evaluate the real-world outcomes of patients with triple-negative breast cancer treated with carboplatin-containing neoadjuvant chemotherapy at a Brazilian tertiary cancer center, focusing on: (1) the rate of pathologic complete response (pCR) following NACT; (2) patterns of non-pathologic complete response (non-pCR); and (3) the impact of adjuvant capecitabine on disease-free survival (DFS) and overall survival (OS) in women with non-pCR after carboplatin-based NACT.

## Subjects and methods.

**Fig. 1:**
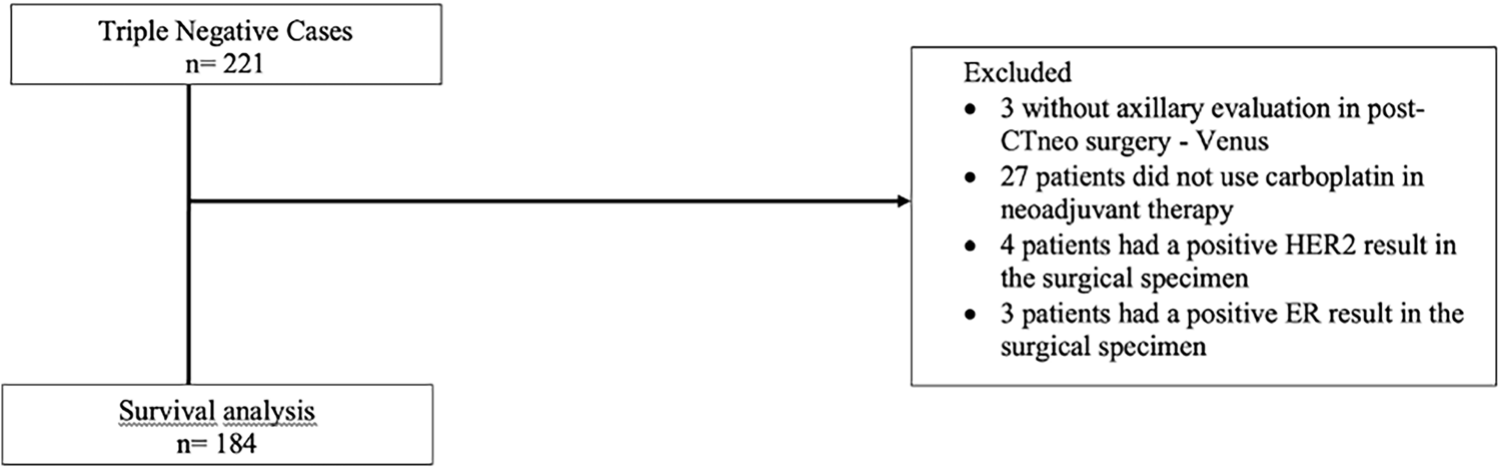
Flowchart. Flowchart of patient selection for survival. From 221 triple-negative breast cancer (TNBC) cases initially identified, 37 were excluded for not meeting inclusion criteria, resulting in 184 cases for survival analysis. All included patients had prior biobank consent; no additional recruitment or refusals occurred for this study

This study is a secondary analysis of the project “H-score and ERBB2 mRNA Refine Identification of HER2-Low and HER2-Ultralow Breast Cancer.” The project was approved by the UNICAMP Research Ethics Committee (CAAE 57159722.3.2002.5404), and tumor samples were obtained from the CAISM Biobank under informed consent. All patients included had previously provided written informed consent through the institutional CAISM Biobank (B-056) at the time of diagnosis and tissue banking. No additional recruitment or consent process was conducted specifically for this secondary analysis, and no patients were approached or declined participation. For this reconstituted cohort, we selected treatment-naïve women with TNBC managed at Hospital da Mulher Prof. Dr. José Aristodemo Pinotti (CAISM/UNICAMP) between March 2017 and December 2023, with follow-up through June 2025, who provided biological material to Biobank after signing informed consent. This was a convenience sample including all women who received NACT followed by surgery. The study did not modify clinical management. Among a reconstituted cohort of 221 women identified, we excluded three without axillary assessment at surgery after NACT [18], twenty-seven who did not receive carboplatin in the neoadjuvant setting, four with HER2-positive results, and three with estrogen-receptor-positive results in the post-NACT surgical specimen. Thus, 184 patients who received carboplatin-based NACT and underwent surgery with assessment of Residual Cancer Burden (RCB) were included (Fig. 1).

We assessed the association between age, menopausal status, stage [19, 20], histologic type, histologic grade, HER2 expression, Ki-67 index, number of chemotherapy cycles, and response to NACT by Residual Cancer Burden [21], classified as pCR or non-pCR. We then evaluated DFS and OS according to NACT response and the association of adjuvant capecitabine among non-pCR cases. Adjuvant capecitabine for women with residual disease in the post-NACT surgical specimen was approved for use at CAISM starting June 2020. The drug was offered to 44 women at the discretion of the treating physician. Follow-up followed institutional routine: every 21 d during NACT, on demand after surgery, quarterly and semiannually thereafter, and was updated in June 2025.

The clinicopathological characteristics of the patients were compared according to the pathological response after neoadjuvant chemotherapy, categorized as pCR (RCB 0) or non-pCR (RCB I–III). Continuous variables were described as mean and standard deviation and compared between groups using the Student’s *t*-test or the Mann-Whitney test, as appropriate. Categorical variables were presented as absolute and relative frequencies and assessed using Pearson’s chi-square or Fisher’s exact test, when applicable. Odds ratios (OR) and corresponding 95% confidence intervals for factors associated with pCR were estimated using univariable logistic regression models with pCR (yes/no) as the dependent variable. The significance level was set at 5% (*p* < 0.05). Analyses of DFS and OS were performed using the Kaplan-Meier method, comparing three groups: patients with pCR, those with residual disease treated with capecitabine, and those with residual disease without capecitabine. Differences between survival curves were tested using the log-rank test and adjusted by the Holm-Šidák method (*p* < 0.05).

Within the non-pCR subgroup, additional exploratory analyses were conducted according to Residual Cancer Burden class, stratifying patients into RCB-I and RCB-II/III. Kaplan–Meier curves, log-rank tests, and Cox proportional hazards models were fitted within each RCB stratum to evaluate the association between adjuvant capecitabine and survival outcomes. Formal interaction between capecitabine exposure and RCB stratum was assessed in exploratory Cox models by including a multiplicative interaction term (capecitabine × RCB stratum). To identify independent prognostic factors in the non-pCR group, multivariable Cox proportional hazards models were fitted. In the primary prognostic models, analyses were stratified by adjuvant capecitabine use, allowing each group to have its own baseline hazard. Covariates were defined a priori based on clinical relevance and included age at diagnosis, menopausal status, clinical TNM stage (I/II/III), histologic subtype, histologic grade on biopsy, HER2 immunohistochemistry classification, and Ki-67 expression on biopsy (continuous). To improve model stability, histologic grade categories with low frequency were collapsed (G1–2 vs G3). Complementary multivariable Cox models were also constructed within the non-pCR subgroup directly estimating the association between adjuvant capecitabine exposure (yes/no) and survival outcomes, adjusting for Residual Cancer Burden class and the same prespecified clinicopathologic covariates. Multivariable analyses were performed using a complete-case approach (listwise deletion). Of the 111 patients in the non-pCR group, 98 had complete data for all prespecified covariates and were included in the overall multivariable models. The proportional hazards assumption was confirmed using the Schoenfeld test (global test: OS *p* = 0.66; DFS *p* = 0.50). Hazard ratios (HR) and their corresponding 95% confidence intervals (95% CI) were calculated. Follow-up maturity was estimated using the reverse Kaplan–Meier method, treating censored observations as events. Overall survival time with inverted event coding (1 - event) was used to estimate median follow-up for the overall cohort and predefined subgroups. To assess the potential impact of differential calendar-time introduction of capecitabine, sensitivity analyses were performed using administrative censoring at 36 and 48 months within the non-pCR subgroup. Follow-up time was truncated at prespecified horizons (T* = min[T, τ]), and events beyond τ were treated as censored. Kaplan–Meier, log-rank tests, and Cox proportional hazards models evaluating the association between capecitabine and survival were re-estimated under these conditions.

## Results

In the cohort, pCR occurred in 73 (40%) women and non-pCR in 111 (60%). Median follow-up estimated by the reverse Kaplan–Meier method was 58.31 months (95% CI 53.25–64.13) for the overall cohort and 64.09 months (95% CI 56.18–68.53) for the non-pCR subgroup. Among non-pCR patients, median follow-up was 41.59 months (95% CI 35.61–51.28) in those receiving capecitabine and 71.68 months (95% CI 67.51–83.77) in those not receiving capecitabine. Table 1 shows clinicopathologic characteristics by pathologic response after NACT. Younger age was significantly associated with pCR (49 vs 52 years). Earlier clinical stages (I-II) were also associated with higher pCR rates (57% vs 36%) as was high histologic grade (grade 3; 88% vs 73%). Menopausal status, histologic type, HER2 expression, and completeness of chemotherapy regimen were not associated with NACT response.

**Table 1:** Characteristic of the women according pathological response after neoadjuvant chemotherapy

Characteristic	Total (*n* %)	pCR	non-pCR	*P* (univariate)	OR (95% CI)
Age (years) mean (SD)	51 (41–61)	49 (39–59)	52 (41–53)	0.03	-
Menopausal status				0.86	1.21 (0.67–2.20)
Pre menopausal	98 (53)	41 (56)	57 (51)		
Post menopausal	86 (47)	32 (44)	54 (49)		
Clinical stage (TNM)				0.03	0.43 (0.23–0.79)
1–2	83 (45)	42 (58)	41 (37)		
3	101 (55)	31 (42)	70 (63)		
Histological type				0.33	2.24 (0.36–13.75)
Special	8 (3)	3 (4)	5 (2)		
No special type	176 (97)	70 (96)	106 (98)		
Histological grade				0.02	NA
Grade 1–2	38 (21)	10 (14)	28 (25)		
Grade 3	146 (79)	63 (86)	83 (75)		
HER2⧫				0.61	NA
Null	61 (37)	28 (38)	33 (30)		
Ultralow	30 (18)	12 (16)	18 (16)		
Low	76 (43)	30 (41)	46 (41)		
Ki-67 § (Mean)					NA
Mean (SD)	61.6 (37.6–85.6)	60.9 (39.0 −82.8)	62.1 (36.8–85.4)	0.93	
Neoadjuvant chemotherapy				0.87	NA
Complete regimen (AC-TCb)	138 (75)	58 (79)	80 (72)		
Incomplete regimen (AC-TCb)	46 (25)	15 (21)	31 (28)		
Surgery				0.88	NA
Mastectomy	83 (45)	32 (44)	51(46)		
Conservative surgery	101 (55)	41 (56)	60 (54)		
Axillary surgery				< 0.001	3.37 (1.79–6.34)
Sentinel lymph node biopsy	85 (46)	21(29)	64(58)		
Axillary lymph node dissection	99(54)	52(71)	47(42)		
Radiotherapy				0.79	NA
No	9 (5)	4 (5)	5 (5)		
Yes	175 (95)	69 (95)	106 (95)		
Total	184 (100%)	73 (40)	111 (60%)		

*pCR* pathologic complete response, non*-pCR* non-pathologic complete response, Residual cancer burden I, II, III.

⧫ HER2 H-Score not performed in 16 cases.

§ Ki-67 not accessible in 1 biopsy. Complete regimen = 4 cycles of anthracycline + cyclophosphamide followed by weekly carboplatin + paclitaxel.

Incomplete regimen =  < 4 cycles of anthracycline + cyclophosphamide or < 12 weeks of carboplatin + paclitaxel

Baseline characteristics of patients with residual disease according to capecitabine exposure are summarized in Table 2. Patients receiving capecitabine were significantly younger (mean 44 vs 56 years; *p* < 0.001), more likely to have completed the full neoadjuvant chemotherapy regimen (81% vs 55%; *p* = 0.007), and more frequently classified as RCB II–III (*p* = 0.016). Clinical stage, histologic grade, HER2 classification, Ki-67 expression, type of surgery, axillary management, and radiotherapy did not differ significantly between groups. These differences indicate that treatment allocation was not random and may reflect physician selection based on residual tumor burden and patient fitness.

**Table 2:** Clinicopathologic characteristics of patients with residual disease (non-pCR) according to adjuvant capecitabine use

Characteristic	Total non-pCR (*n *= 111)	Capecitabine (*n *= 44)	No capecitabine (*n* = 67)	*P* (univariate)
Age (years) Mean (SD)	51 (40–63)	44 (37–51)	56 (46–66)	< 0.001
Menopausal status				0.88
Pre menopausal	77(69)	21 (47)	33 (49)	
Post menopausal	34(31)	23 (53)	34 (51)	
Clinical stage (TNM)				0.21
1–2	41 (37)	19 (44)	22 (32)	
3	70 (63)	25 (56)	45 (68)	
Histological type				0.15
Special	5 (5)	-	5 (8)	
No special type	106 (95)	44 (100)	62 (92)	
Histological grade				0.65
Grade 1–2	28 (26)	10 (23)	18 (27)	
Grade 3	83 (74)	34 (77)	49 (73)	
HER2⧫				0.35
Null	34 (30)	16 (16)	18 (26)	
Ultralow	18 (16)	9 (20)	9 (13)	
Low	46 (41)	16 (36)	30 (44)	
Ki-67 § (Mean)				0.62
Mean (SD)	64.8 ± 25.7	63.2 ± 28.4	65.9 ± 23.8	
Neoadjuvant chemotherapy				0.007
Complete regimen (AC-TCb)	73 (65)	36 (81)	37 (55)	
Incomplete regimen (AC-TCb)	38 (35)	8 (19)	30 (45)	
RCB				0.016
I	20 (18)	3 (7)	17	
II	69 (62)	34 (77)	35 (52)	
III	22 (20)	7 (16)	15 (22)	
Surgery				0.98
Mastectomy	55 (49)	22 (50)	33 (49)	
Conservative surgery	56 (51)	22 (50)	34 (51)	
Axillary surgery				0.96
Sentinel lymph node biopsy	47 (42)	19 (43)	28 (42)	
Axillary lymph node dissection	64 (58)	25 (57)	39 (58)	
Radiotherapy				1.00
No	5 (5)	2 (5)	3 (4)	
Yes	106 (95)	42 (95)	64 (96)	
Total	111 (100)	44 (40)	67 (60)	

Non-*pCR* non-pathologic complete response; Residual cancer burden I, II, III.

⧫ HER2 H-Score not performed in 13 cases, excluded from analysis.

§ Ki-67 not accessible in 1 biopsy. Complete regimen = 4 cycles of anthracycline + cyclophosphamide followed by weekly carboplatin + paclitaxel.

Incomplete regimen =  < 4 cycles of anthracycline + cyclophosphamide or < 12 weeks of carboplatin + paclitaxel

Among patients with residual disease after neoadjuvant chemotherapy, 44 received adjuvant capecitabine. Of these, 29 (65.9%) completed the planned eight cycles, whereas 15 (34.1%) received fewer than eight cycles. The median number of capecitabine cycles administered was eight, ranging from one to eight cycles.

**Fig. 2:**
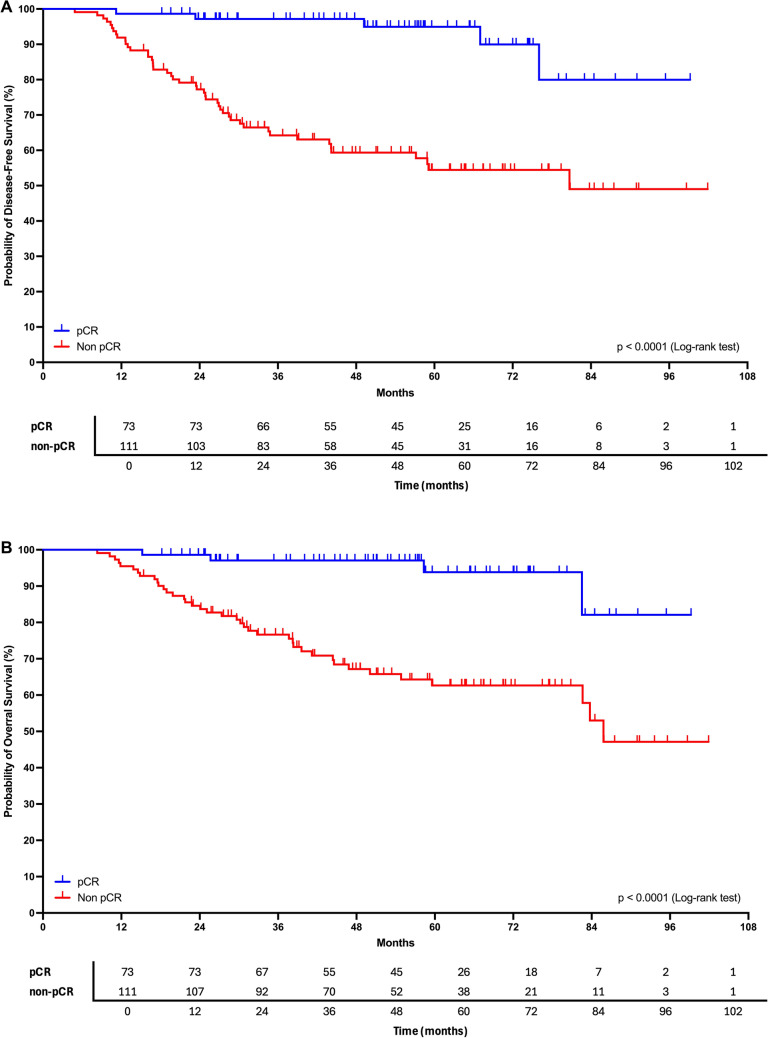
**A** Kaplan–Meier disease-free survival curves comparing patients with pCR versus non-pCR after neoadjuvant chemotherapy. DFS was significantly longer among women with pCR (log-rank p < 0.0001), and median DFS was not reached. **B** Kaplan–Meier overall survival curves comparing patients with pCR versus non-pCR. OS was significantly longer among women achieving pCR (log-rank p < 0.0001), with no median reached in the pCR group

In survival analyses, pCR was strongly associated with improved outcomes. For disease-free survival (DFS), 51 events occurred among the 184 women, and Kaplan–Meier curves demonstrated marked separation between groups, with significantly longer DFS among women who achieved pCR (log-rank *p* < 0.0001). Median DFS was not reached in the pCR group. Overall survival (OS) analysis yielded similar results: among 43 deaths (41 disease-related), patients with pCR showed significantly longer survival, with no median OS reached, whereas non-pCR patients experienced earlier and more frequent events (log-rank *p* < 0.0001). In univariable Cox models, having residual disease was associated with a nearly sevenfold higher risk of death (HR = 6.98; 95% CI 2.49–19.55; *p* = 0.0002) and recurrence (HR = 7.17; 95% CI 2.85–18.05; *p* < 0.0001). Schoenfeld residuals indicated no violation of proportional hazards. Fig. 2.

**Fig. 3:**
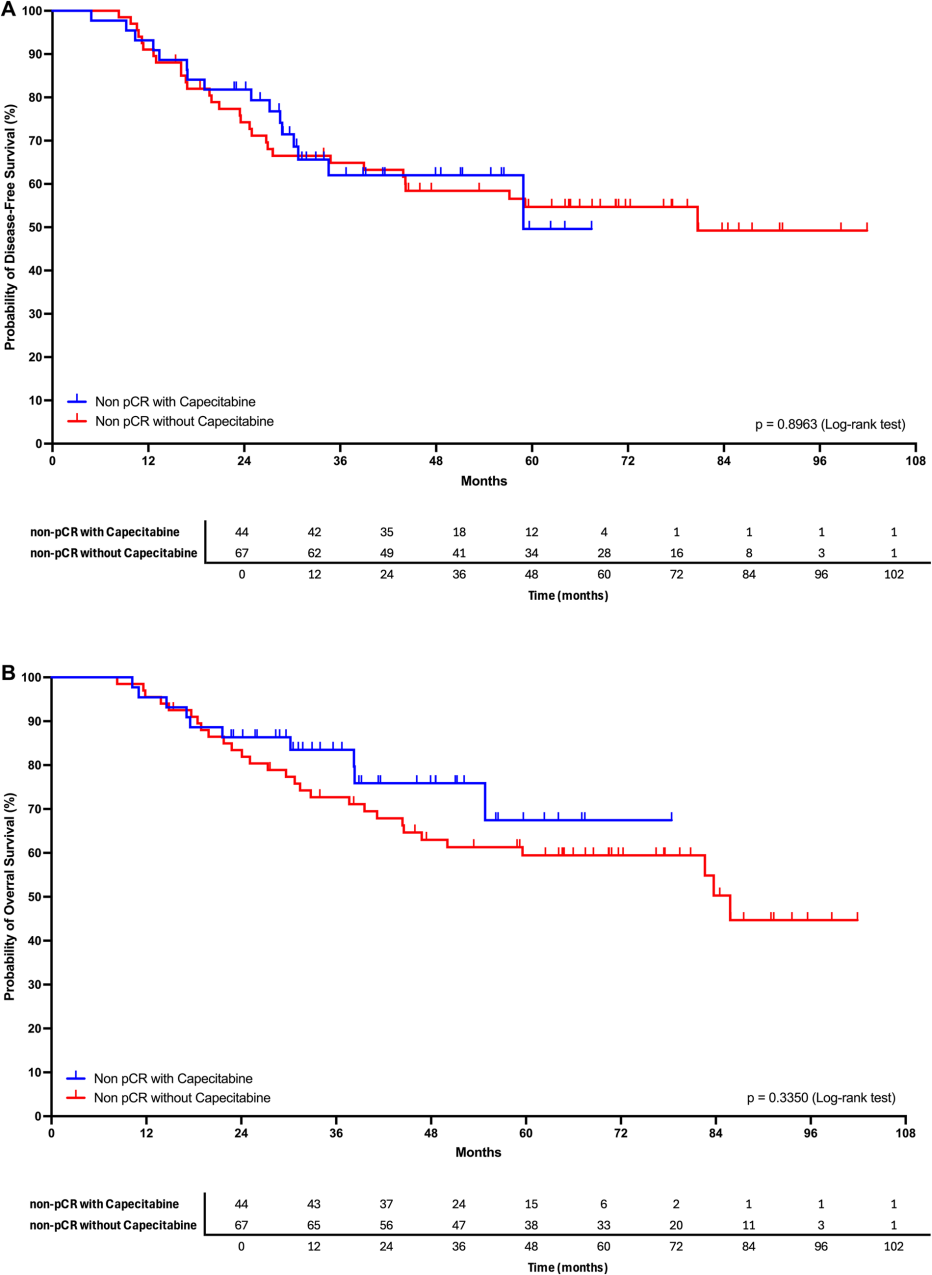
**A** Kaplan–Meier disease-free survival curves among patients with residual disease (non-pCR), comparing those treated with adjuvant capecitabine versus those who did not receive the drug. No significant difference was observed between groups (log-rank *p* ≈ 0.95; HR = 0.96; 95% CI 0.52–1.78). **B** Kaplan–Meier overall survival curves among patients with residual disease (non-pCR) according to capecitabine use. Curves were largely overlapping, with no significant difference (log-rank *p* ≈ 0.34; HR = 0.70; 95% CI 0.33–1.46)s

Among the 111 women with residual disease, 44 received adjuvant capecitabine and 67 did not. DFS curves for these two groups were nearly superimposed, with no consistent divergence throughout follow-up. The log-rank test (p ≈ 0.95) and the Gehan–Breslow–Wilcoxon test (*p* ≈ 0.90) both showed no significant difference. Cox univariate analysis confirmed the absence of effect (HR = 0.96; 95% CI, 0.52–1.78; *p* = 0.90). OS results were consistent, with extensively overlapping curves between capecitabine-treated and untreated patients; log-rank *p* ≈ 0.34; HR = 0.70 (95% CI, 0.33–1.46; *p* = 0.34). Median OS was not reached in the capecitabine group and was approximately 86 months among women who did not receive the drug, with wide confidence intervals. Fig 3

Sensitivity analyses using administrative censoring at 36 and 48 months yielded consistent results. For DFS, hazard ratios remained close to unity after truncation (HR 0.9966 at 36 months; HR 0.8996 at 48 months), with no significant differences observed. For OS, point estimates remained numerically favorable toward capecitabine but imprecise (HR 0.6090 at 36 months; HR 0.6424 at 48 months), with overlapping confidence intervals. These findings indicate that differential follow-up duration did not materially influence treatment effect estimates.

Exploratory analyses stratified by Residual Cancer Burden were then performed. Among non-pCR patients, 20 were classified as RCB-I and 91 as RCB-II/III. In RCB-I, event rates were low, and no significant association between capecitabine and DFS or OS was observed. In RCB-II/III, capecitabine was not significantly associated with DFS (HR = 0.86; 95% CI 0.45–1.63; *p* = 0.65) or OS (HR = 0.65; 95% CI 0.30–1.40; *p* = 0.27) in univariable models. Multivariable models within RCB-II/III yielded similar results (DFS HR = 0.87; 95% CI 0.28–2.69; *p* = 0.81; OS HR = 0.42; 95% CI 0.12–1.47; *p* = 0.17). Formal interaction testing between capecitabine and RCB stratum produced unstable estimates due to sparse events in the RCB-I capecitabine subgroup and did not demonstrate statistically significant effect modification.

To better characterize prognostic factors among women with residual disease, multivariable Cox models stratified by capecitabine use were constructed. Stratification allowed each treatment group to retain its own baseline hazard while estimating independent associations of clinicopathologic variables with survival. Because of sparse data in specific categories, histologic grade was grouped into G1–2 versus G3, and HER2 expression into null versus low/ultra-low. After excluding patients with missing data, 98 women contributed to the multivariable models. For OS, the global model was statistically significant (*χ*^2^ = 8.19; *p* = 0.042), with Ki-67 emerging as the only independent prognostic factor (HR = 1.03 per 1% increase; 95% CI, 1.00–1.05; *p* = 0.020). For DFS, the multivariable model was not significant (*χ*^2^ = 4.48; *p* = 0.21), although Ki-67 demonstrated a borderline association with recurrence (HR = 1.02; 95% CI, 0.999–1.04; *p* = 0.061). Complementary multivariable models directly including capecitabine exposure as a covariate, adjusted for RCB and clinicopathologic factors, yielded results consistent with the primary analyses. Capecitabine was not independently associated with DFS, and OS estimates remained numerically favorable but imprecise, with wide confidence intervals crossing unity. Table 3.

**Table 3:** Cox proportional hazards models for overall survival (OS) and disease-free survival (DFS) in patients with non-pCR, according to capecitabine use and clinicopathologic covariates

Variable	HR (IC95%)	p	HR (IC95%)	p
	Univariate DFS		Univariate OS	
non-pCR with capecitabine vs without	0.96 (0.52–1.78)	0.895	0.70 (0.33–1.46)	0.337
	Multivariable DFS*		Multivariable OS*	
Age at diagnosis (years)	1.01 (0.97–1.05)	0.819	1.01 (0.97–1.06)	0.531
Menopausal status (pre vs post)	1.62 (0.86–3.05)	0.133	1.93 (0.94–3.96)	0.073
TNM II vs. III	3.26E + 07 (0–∞)	0,997	1.71E + 07 (0–∞)	0.998
TNM III vs. I	4.80E + 07 (0–∞)	0,997	3.81E + 07 (0–∞)	0.998
Histologic subtype (others vs ref.)	0.54 (0.11—2.53)	0.432	0.31 (0,04–2,59)	0.278
Histologic grade 3 vs. 1–2	0.69 (0.26–1.85)	0.458	0.71 (0.25–2.02)	0.519
HER2 Null vs. low	1.15 (0.25–5.31)	0.858	1.75 (0.31–9.77)	0.523
HER2-Ultralow vs. low	0.43 (0.16–1.15)	0.093	0.48 (0.15–1.52)	0.213
Ki-67 (continuous,%)	1.03 (1.00–1.05)	0.02	1.03 (1.00–1.04)	0.066

*HR* hazard ratio, 95% CI 95% confidence interval. *Multivariable models stratified by group (non-pCR with vs without capecitabine).

Variables without identifiable estimates were omitted

In summary, pCR emerged as the strongest prognostic factor in this real-world cohort. Among patients with residual disease, adjuvant capecitabine was not associated with statistically significant improvement in DFS or OS, and no clear evidence of effect modification by residual disease burden was observed.

## Discussion

We evaluated the role of adjuvant capecitabine in oncologic outcomes among women with TNBC previously treated with carboplatin-containing NACT. Younger age, earlier stage and higher histologic grade were associated with higher pCR rates, consistent with increased chemosensitivity in highly proliferative tumors. DFS and OS were directly related to achieving pCR, with only four disease-related deaths in that group. Higher protein Ki-67 expression was independently associated with worse DFS and OS, reinforcing its role as a prognostic biomarker of proliferative activity.

The pCR rate observed here (40%)-achieved with weekly carboplatin AUC2-was similar to rates reported in large trials adding carboplatin to NACT and significantly increasing pCR compared with anthracycline-taxane regimens alone. In the meta-analysis by Pathak et al. (2022), adding carboplatin in the neoadjuvant setting more than doubled pCR odds (OR 2.11; 95% CI 1.44–3.08; *p* = 0.009) [8]. Across 1,635 women receiving neoadjuvant carboplatin and 790 treated up front with adjuvant carboplatin, the agent was significantly associated with better DFS (HR 0.66; 95% CI 0.55–0.80; *p *< 0.001) and OS (HR 0.68; 95% CI 0.54–0.87; *p* = 0.002). Regarding the optimal platinum regimen in NACT, Petrelli et al. (2024) reported that carboplatin AUC 5 was associated with the greatest benefit in terms of pCR (RR 2.23; 95% CI 1.60–3.20) and disease-free survival (HR 0.36; 95% CI 0.18–0.73) compared with other platinum schedules [22]. For over a decade, pCR has been the best prognostic marker in TNBC [9;24].

Histologic grade was significantly associated with higher pCR rates, with poorly differentiated tumors showing greater chemosensitivity, likely due to higher proliferation and genomic instability [21]. CTNeoBC data indicate grade III tumors have pCR in 35–40%, whereas grade I-II rarely exceed 15–20% [24]. However, grade did not translate into significant DFS/OS effects in our cohort, aligning with and subsequent analyses [23, 25], where grade is not an independent long-term prognostic factor after adjusting for pCR.

Higher pCR rates were observed in earlier clinical stages, consistent with literature. [26] reported pCR 47% in stage II and 19% in stage III with standard NACT without carboplatin [26]. Recently, NeoPACT [27] reported pCR 69, 58, and 43% in stages I, II, and III, respectively, with carboplatin plus pembrolizumab. In KEYNOTE-522, adding pembrolizumab to a carboplatin backbone yielded higher pCR in earlier stages (IIA-IIB: 68–70% vs 54–56% control) than in stage III (50–55 vs 40%), although carboplatin’s relative benefit was present across subgroups [2–4]. Importantly, while achieving pCR is consistently associated with improved long-term outcomes, emerging data suggest that baseline clinical stage may still retain prognostic relevance even among patients who achieve pCR. Studies such as those by Huober et al. and Boman et al. demonstrated that initial tumor burden and stage at diagnosis can remain independently associated with relapse risk despite pCR, indicating that pCR does not entirely abrogate the adverse prognostic impact of more advanced disease [28, 29].

Beyond individual trials, recent pooled evidence has further clarified the impact of neoadjuvant carboplatin on response and survival outcomes in early-stage TNBC. A pooled analysis of the BrighTNess, CALGB 40603 (Alliance), and GeparSixto randomized trials (*n* = 1,084) demonstrated that the addition of carboplatin to neoadjuvant chemotherapy significantly increased pCR rates (OR 1.89; 95% CI 1.41–2.55; *p* < 0.001) and improved event-free survival (EFS) (HR 0.71; 95% CI 0.54–0.93; *p* = 0.01), although no overall survival (OS) benefit was observed (HR 0.93; 95% CI 0.65–1.31; *p* = 0.66) [30]. Notably, among germline BRCA1/2 mutation carriers, carboplatin was associated with a borderline EFS benefit despite no significant impact on pCR or OS. In addition, immune-related gene expression signatures were consistently prognostic for pCR and survival outcomes, but none demonstrated a predictive interaction with carboplatin benefit. Collectively, these data reinforce the central role of carboplatin in modern neoadjuvant strategies for TNBC, particularly in the post-KEYNOTE-522 era, while highlighting pCR and tumor biology, rather than treatment intensification alone, as the main drivers of long-term prognosis. Importantly, during the study period (2017–2020), neoadjuvant immunotherapy was not routinely available in the Brazilian public health system. Therefore, this cohort reflects a predominantly pre-immunotherapy real-world scenario, which remains clinically relevant in middle-income settings where access to immune checkpoint inhibitors can still be limited.

In our study, both DFS and OS were significantly longer among women with pCR. This suggests that covariate effects on recurrence risk may not be constant over time-possibly reflecting TNBC’s characteristic early relapse pattern concentrated in the initial years after treatment. Hence, part of the observed association between clinical factors and DFS may be restricted to this early high-risk period. DFS and OS curves showed markedly better prognosis in pCR versus non-pCR, reinforcing pCR as a robust prognostic marker in TNBC. However, among non-pCR patients, adjuvant capecitabine was not associated with statistically significant improvement in DFS or OS in unadjusted analyses, and this finding remained consistent after multivariable adjustment. This contrasts with some randomized trials but aligns with other clinical trials and a growing segment of real-world evidence suggesting heterogeneity of benefit in this context. Exploratory analyses stratified by Residual Cancer Burden did not demonstrate clear heterogeneity of treatment effect. Although point estimates in the RCB-II/III subgroup numerically favored capecitabine, confidence intervals were wide and crossed unity. Formal interaction testing between capecitabine and RCB class was limited by sparse events, particularly in the RCB-I capecitabine subgroup, resulting in unstable estimates. Therefore, no definitive evidence of effect modification by residual tumor burden could be established in this cohort.

In our real-world Brazilian cohort, adjuvant capecitabine was not associated with statistically significant improvement in survival outcomes following carboplatin-containing NACT. These findings align with negative real-world studies, reinforcing that although capecitabine is an established option after residual disease, its additional impact after prior platinum exposure remains uncertain. Variability across studies suggests that clinical, biological, and therapeutic factors-such as residual tumor burden and prior platinum-may modulate the true benefit in this subgroup. Possible explanations for the lack of difference in our cohort include the small number of events, limited statistical power, and selection biases. Thus, in this patient set, residual disease after NACT remained the dominant prognostic determinant, and any incremental benefit from capecitabine could not be demonstrated within the limits of this study.

In the literature, CREATE-X’s TNBC subgroup showed a pronounced benefit of capecitabine (3-year DFS 69.8 vs 56.1%; HR 0.58; 95% CI 0.39–0.87; and 3-year OS 78.8 vs 70.3%; HR 0.52; 95% CI 0.30–0.90) [10]. SYSUCC-001 (maintenance capecitabine) similarly improved survival [11]. In contrast, the GEICAM/CIBOMA trial was conducted in a different clinical context, enrolling patients who had completed standard (neo)adjuvant chemotherapy and not specifically restricted to those with residual disease after neoadjuvant treatment. Consequently, its design differs from CREATE-X and from the present study, which specifically focus on the post-neoadjuvant residual disease setting [12]. These differences in study population and design may partly explain the heterogeneity observed across randomized trials.

This contrasts with the positive results of the CREATE-X trial [10] which established adjuvant capecitabine as a post-neoadjuvant option in TNBC. However, several methodological limitations restrict its applicability to patients previously exposed to platinum-containing regimens. CREATE-X enrolled only Asian women-whose pharmacogenomic profile may enhance capecitabine efficacy-and tested the drug after non-platinum chemotherapy, unlike current TNBC standards. Because both platinum agents and fluoropyrimidines act through DNA-damage mechanisms, sequential use might limit additive benefit once maximal cytotoxic effect is achieved. The small TNBC subgroup, modest treatment adherence, and absence of biomarkers such as Residual Cancer Burden further limit the study’s external validity in modern practice. These differences may help contextualize the absence of statistically significant association observed in our cohort. In a platinum-treated real-world population, any additional benefit of capecitabine may be attenuated or more difficult to demonstrate. Dedicated trials are needed to clarify its role in the post-platinum setting, where TNBC management has evolved beyond the CREATE-X era.

Real-world studies are also heterogeneous. The single-arm Italian CaRe study (270 TNBC with residual disease after NACT) reported 2-year DFS 62 and OS 84%, comparable to CREATE-X; however, NACT predominantly used anthracyclines/taxanes, and only 4.1% (11 patients) received platinum-based NACT, limiting comparisons with carboplatin-treated populations [14]. In a Korean real-world study including 478 patients with residual TNBC after neoadjuvant chemotherapy [15], 402 received adjuvant capecitabine and 73 did not receive additional systemic therapy. Among the overall cohort, 126 patients had previously received platinum-based neoadjuvant chemotherapy. Adjuvant capecitabine was associated with improved distant DFS (3-year 86.3 vs 74.4%; HR 0.53; 95% CI 0.31–0.91; *p* = 0.019) and OS (93.3% vs 83.8%; HR 0.49; 95% CI 0.25–0.95; *p* = 0.032). In the platinum-NACT subgroup, capecitabine reduced the risk of distant recurrence and death by 81% (HR 0.19; 95% CI 0.04–0.89; *p* = 0.035), supporting potential benefit in this setting [15]. By contrast, a Latin American real-world study from Colombia (*n* = 360 TNBC with residual disease; 106 capecitabine) found only 31% had platinum-based NACT (more frequent in the capecitabine group, 60 vs 19%). After propensity score matching (*n* = 187; 72 capecitabine vs 115 controls), there was no overall benefit in OS (HR 0.79; 95% CI 0.51–1.23; *p* = 0.302; medians 82.4 vs 52.1 months) or DFS (HR 0.81; 95% CI 0.53–1.23; *p* = 0.321; medians 62.6 vs 32.2 months). However, stratified analyses showed significant benefit in patients with high residual tumor burden (ypT3-T4: OS HR (HR 3.14, 95% CI 1.81–5.44, *p* < 0.001) and DFS (HR 1.96, 95% CI 1.22–3.15, *p* = 0.005), and in those not receiving radiotherapy (DFS HR 0.47; 95% CI 0.23–0.96; *p* = 0.038), suggesting drug effect may be modulated by clinical/therapeutic factors [16]. Similarly, a Brazilian real-world cohort from Ribeirão Preto evaluated 153 TNBC patients treated with standard anthracycline- and taxane-based NACT, reporting a pCR rate of 34.6% and showing that patients achieving pCR had significantly improved 5-year disease-specific survival (HR 0.078; 95% CI 0.02–0.32; *p* < 0.0001). Among the 100 patients with residual disease, 41% received adjuvant capecitabine; however, no survival benefit was observed with its addition (HR 0.9; 95% CI 0.4–1.8; *p* = 0.7), despite the cohort being enriched for high-risk features, including stage III disease in 58% and high-grade tumors in 61% of cases [17]. Taken together, these real-world data highlight substantial heterogeneity in the observed benefit of adjuvant capecitabine and suggest that its effect may be strongly modulated by prior systemic therapy, residual tumor burden, and clinical context.

Although Ki-67 was not significantly associated with pCR in this cohor, it retained independent prognostic significance for DFS but not OS among patients with residual disease, with each unit increase associated with worsening outcomes. These findings are consistent with the literature: In a meta-analysis including 35 studies with 7,716 patients, high Ki-67 expression was significantly associated with worse DFS (HR = 1.73, 95% CI 1.45–2.07, *p* < 0.001) and OS (HR = 1.65, 95% CI 1.27–2.14, *p* < 0.001) among patients with resected TNBC. In the subgroup analysis, when a cutoff of Ki-67 ≥ 40% was applied, the pooled HRs for DFS and OS were 2.30 (95% CI 1.54–3.44, *p* < 0.001) and 2.95 (95% CI 1.67–5.19, *p* < 0.001), respectively, reinforcing the negative prognostic impact of high proliferative activity in TNBC [31]. Thus, although high Ki-67 is associated with higher pCR rates, it also predicts worse long-term prognosis when pCR is not achieved—likely reflecting that highly proliferative tumors which fail to respond to chemotherapy retain aggressive biological features.

Limitations of this study include its retrospective design and the limited number of events, particularly in the pCR group (only four deaths), which reduces the precision of survival estimates and results in high hazard ratios with wide confidence intervals. The study was not powered to detect moderate survival differences between treatment groups; therefore, lack of statistical significance should not be interpreted as evidence of absence of a clinically meaningful effect.

There is potential confounding by indication when comparing patients with residual disease who did and did not receive capecitabine. In our cohort, patients receiving capecitabine were younger and more frequently presented with higher residual tumor burden (RCB II–III), suggesting physician-driven treatment allocation. Although multivariable adjustment was performed, residual confounding cannot be excluded, as unmeasured clinical factors may have influenced therapeutic decisions and subsequent outcomes.

Additionally, because capecitabine was introduced later during the study period, differences in follow-up duration could theoretically influence survival comparisons. However, sensitivity analyses using administrative censoring at 36 and 48 months yielded hazard ratio estimates consistent with the primary analyses, indicating that unequal follow-up did not materially affect treatment effect estimates in this cohort.

Despite these limitations, our findings are consistent with the literature in confirming pCR as a robust prognostic marker in TNBC. Nevertheless, the incremental benefit of adjuvant capecitabine following prior carboplatin exposure remains uncertain.

## Conclusion

Survival analyses consistently demonstrated that achieving pCR was associated with substantially improved DFS and OS. In multivariable models, higher tumor Ki-67 expression remained an independent marker of poorer survival among patients with residual disease, underscoring the prognostic relevance of proliferative activity. In this real-world cohort treated with carboplatin-based neoadjuvant chemotherapy, adjuvant capecitabine was not associated with statistically significant improvement in survival outcomes. Residual disease burden remained the principal determinant of prognosis, and the potential incremental benefit of capecitabine after prior platinum exposure warrants further investigation in adequately powered studies.

## Data Availability

The datasets generated and/or analyzed during the current study include information obtained from the CAISM/UNICAMP Biobank and therefore cannot be made publicly available due to institutional policies governing data use and protection. De-identified data may be provided by the corresponding author upon reasonable request and in accordance with Biobank regulations and Ethics Committee approval.
